# *In Vitro* Activity of Cefiderocol against U.S. and European Gram-Negative Clinical Isolates Collected in 2020 as Part of the SENTRY Antimicrobial Surveillance Program

**DOI:** 10.1128/spectrum.02712-21

**Published:** 2022-03-09

**Authors:** Dee Shortridge, Jennifer M. Streit, Rodrigo Mendes, Mariana Castanheira

**Affiliations:** a JMI Laboratories, North Liberty, Iowa, USA; Institute of Biomedical Sciences, Universidade de São Paulo

**Keywords:** cefiderocol, activity, surveillance, Gram-negative bacteria

## Abstract

Cefiderocol is a siderophore-conjugated cephalosporin with broad activity against Gram-negative (GN) bacteria, including carbapenem-resistant *Enterobacterales* (CRE), Pseudomonas aeruginosa, Acinetobacter spp., and Stenotrophomonas maltophilia. Cefiderocol was approved by the FDA for treatment of complicated urinary tract infection, hospital-acquired bacterial pneumonia, and ventilator-associated bacterial pneumonia and by the European Medicines Agency (EMA) for aerobic GN infections in adults with few treatment options. In this study, we analyzed the susceptibility of cefiderocol against GN clinical isolates that were collected from hospitalized patients in the United States and Europe in 2020 as part of the SENTRY Antimicrobial Surveillance Program. GN isolates, including 8,047 *Enterobacterales*, 2,282 P. aeruginosa, 650 Acinetobacter species, and 338 S. maltophilia isolates, were consecutively collected from patients in 66 hospitals in 19 countries. Susceptibility testing was performed using the CLSI broth microdilution method, and cefiderocol was tested in iron-depleted cation-adjusted Mueller-Hinton broth. Cefiderocol activity against resistant isolates, including CRE and extensively drug-resistant (XDR) isolates, was determined. *Enterobacterales* susceptibility to cefiderocol was 99.8% (CLSI), and CRE susceptibility was 98.2%. Cefiderocol was the most active antimicrobial against all P. aeruginosa isolates with MIC_50/90_ values of 0.12/0.5 mg/L, respectively (99.6% susceptible). A total of 256 P. aeruginosa isolates were XDR, 97.3% were susceptible to cefiderocol, and 7.4% were susceptible to meropenem. Acinetobacter susceptibility to cefiderocol was 97.7%. S. maltophilia susceptibility to cefiderocol was 100.0% (CLSI, 2021) and 97.9% (CLSI, 2022). These *in vitro* data suggest that cefiderocol is an important therapeutic option for the treatment of infections caused by Gram-negative pathogens, including isolates resistant to carbapenems with few therapeutic options.

**IMPORTANCE** Cefiderocol is the first siderophore-conjugated cephalosporin approved for use in the treatment of human bacterial infections. Cefiderocol has broad-spectrum Gram-negative activity against difficult-to-treat bacterial pathogens that can cause serious infections. Our study examines the activity of cefiderocol against a large global collection of Gram-negative clinical isolates collected from hospitalized patients in 2020. In addition, we compare the activities of cefiderocol and recently approved β-lactam–β-lactamase inhibitor combinations against various antimicrobial-resistant pathogen groups including carbapenem-resistant *Enterobacterales*, meropenem-resistant Pseudomonas aeruginosa, and meropenem-resistant Acinetobacter spp. as well as isolates resistant to most classes of antimicrobial drugs. Cefiderocol was the most active antimicrobial tested against the isolates in this study. Our *in vitro* data suggest that cefiderocol may be useful for treatment of serious infections caused by drug-resistant Gram-negative organisms for patients with limited treatment options.

## INTRODUCTION

Antibiotic resistance is increasing, particularly in Gram-negative species, and has been declared a serious problem by the World Health Organization and the U.S. Centers for Disease Control (1, 2). As carbapenems are frequently used to treat multidrug-resistant pathogens, carbapenem resistance has increased subsequently, particularly in difficult-to-treat organisms such as Klebsiella and Acinetobacter (3, 4). Several β-lactam–β-lactamase inhibitors (BL-BLIs), including meropenem-vaborbactam, imipenem-relebactam, and ceftazidime-avibactam, were developed to treat infections caused by carbapenem-resistant *Enterobacterales* (CRE) and Pseudomonas aeruginosa isolates that produce serine carbapenemases. However, none of these inhibitors has activity against CRE producing metallo-β-lactamases or carbapenem-resistant Acinetobacter (5).

Cefiderocol is a siderophore-conjugated cephalosporin with broad activity against Gram-negative bacteria, including carbapenem-resistant isolates of *Enterobacterales*, Pseudomonas, Acinetobacter, and *Stenotrophomonas* (6, 7). The siderophore enables a novel mechanism of bacterial cell entry via the iron transport system while the cephalosporin nucleus is stable to most β-lactamases and carbapenemases, including metallo-β-lactamases (8, 9). These characteristics allow cefiderocol to remain active against extensively drug-resistant (XDR) isolates, including those resistant to carbapenems, and to β-lactam–β-lactamase inhibitor (BL-BLI) combinations such as ceftazidime-avibactam.

Cefiderocol was recently approved by the European Medicines Agency (EMA) for the treatment of infections caused by Gram-negative bacteria in adult patients with limited treatment options and by the Food and Drug Administration (FDA) for complicated urinary tract infection, hospital-acquired bacterial pneumonia, and ventilator-associated bacterial pneumonia (10, 11).

In this study, we analyzed the susceptibility of cefiderocol and recent BL-BLI combinations against recent Gram-negative isolates, including *Enterobacterales*, P. aeruginosa, Acinetobacter baumannii*-calcoaceticus* complex, and Stenotrophomonas maltophilia, collected from hospitalized patients in the United States and Europe in 2020 as part of the SENTRY Antimicrobial Surveillance Program.

## RESULTS

**FIG 1:**
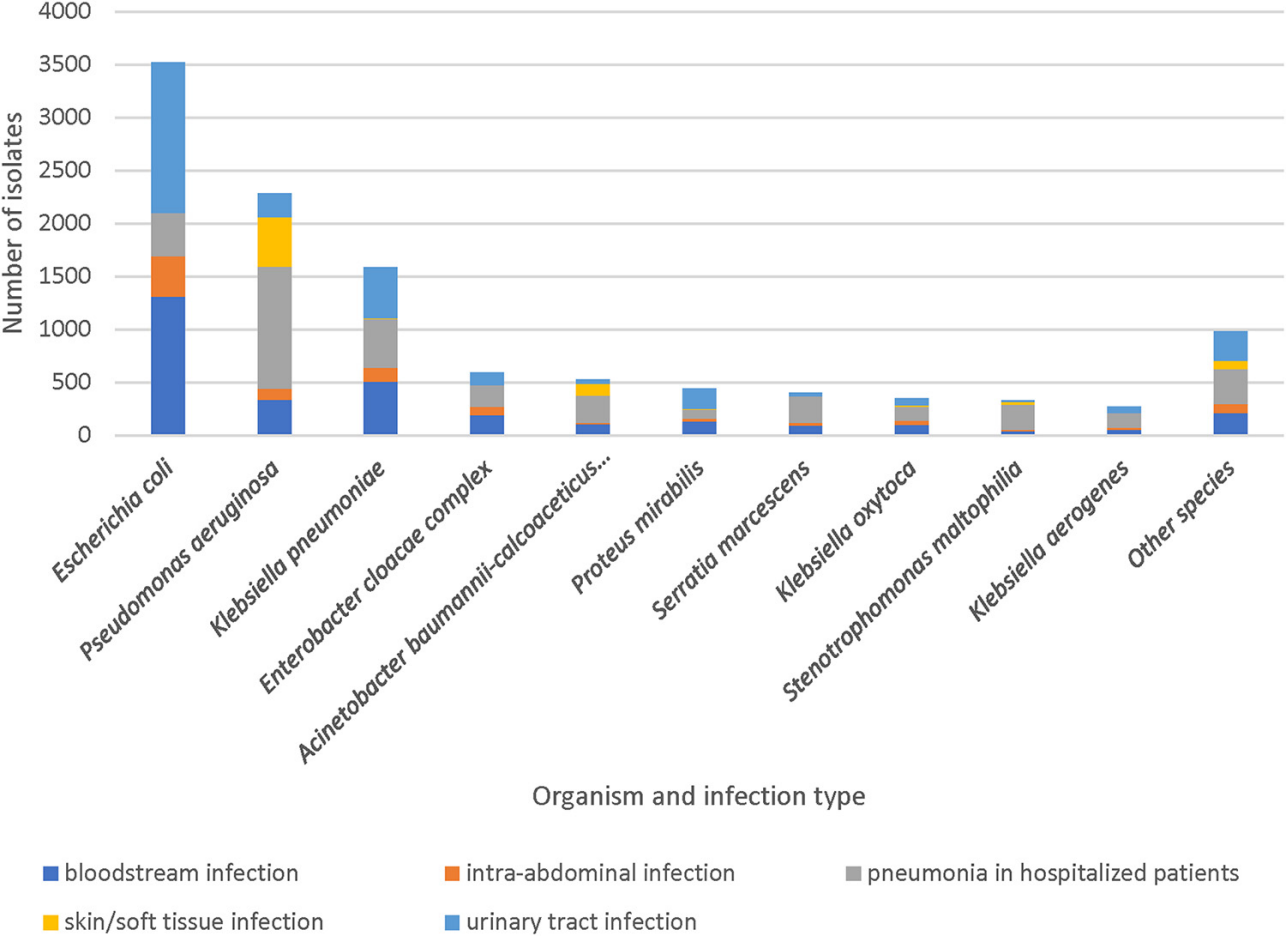
Top 10 species from each infection type.

The most common Gram-negative organism was Escherichia coli (*n *= 3,524) followed by P. aeruginosa (*n *= 2,282), and Klebsiella pneumoniae (*n *= 1,614) (Fig. 1). Isolates were from pneumonia in hospitalized patients (*n *= 3,639), bloodstream infection (*n *= 3,079), urinary tract infection (*n *= 2,923), intra-abdominal infection (*n *= 928), and skin and skin structure infection (*n *= 717). Isolates were evenly distributed between the United States (*n *= 5,702) and Europe (*n *= 5,731).

### *Enterobacterales*.

**FIG 2:**
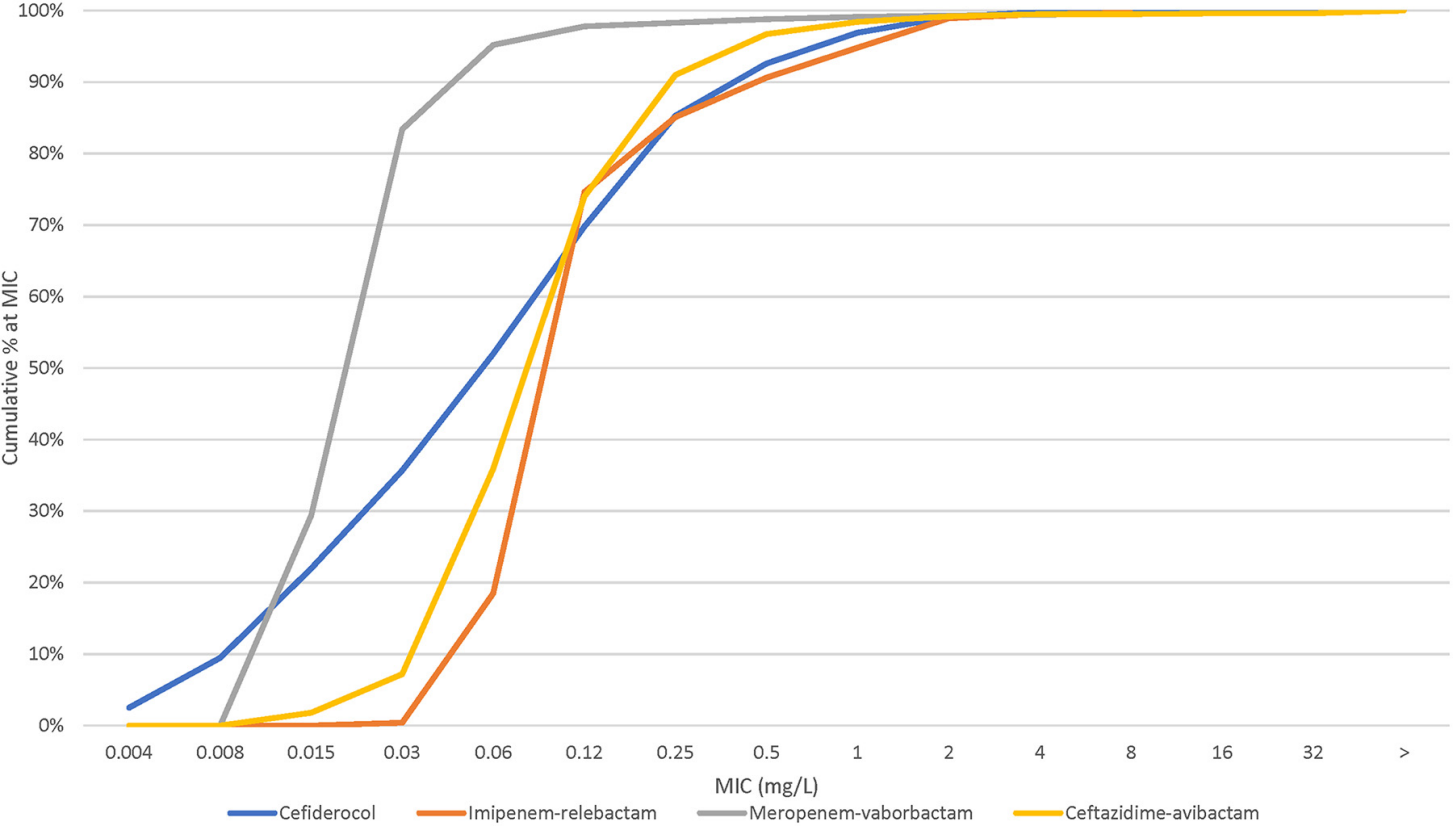
Cumulative percent MIC distribution of cefiderocol and comparators against *Enterobacterales* isolates (*n *= 8,047). >, greater than highest dilution tested.

The susceptibilities based on CLSI criteria (Table 1) and MIC_50/90_ values of cefiderocol and comparators for *Enterobacterales* isolates and isolate groups are shown below (see Table 2). Susceptibilities based on EUCAST and FDA criteria are shown in Table S2 in the supplemental material. The cumulative percent MIC distributions of cefiderocol and key comparators are shown in Fig. 2. Cefiderocol susceptibility was 99.8% (MIC_50/90_, 0.06/0.5 mg/L, respectively). The susceptibilities to the tested comparator agents were >94% against all *Enterobacterales* isolates except for piperacillin-tazobactam (89.0%).

**TABLE 1:** Cefiderocol breakpoints used in this work by organization or agency and organism group

Organism	Breakpoint (mg/L) by organization or agency^*a*^		
	CLSI	FDA	EUCAST
*Enterobacterales*	≤4/8/≥16	≤4/8/≥16	≤2/−/>2
Pseudomonas aeruginosa	≤4/8/≥16	≤1/2/≥4	≤2/−/>2
Acinetobacter species	≤4/8/≥16	≤1/2/≥4	≤2/−/>2^*b*^
Stenotrophomonas maltophilia	≤4/8/≥16 (2021); ≤1/−/− (2022)	NA	≤2/−/>2^*b*^

a Susceptible/intermediate/resistant. NA, not available.

b EUCAST non-species-specific pharmacokinetic/pharmacodynamic (PK/PD) breakpoints used.

For isolates with the CRE phenotype, cefiderocol was the most active agent tested (MIC_50/90_, 0.5/4 mg/L) (see Table 2). The overall CRE rate was 2.1%. A total of 81% (137/169) of the CRE were K. pneumoniae. Cefiderocol had the highest percent susceptibility against CRE (98.2%, CLSI) compared to the BL-BLI combinations tested, for which susceptibilities ranged from 63.9% for imipenem-relebactam to 81.7% for ceftazidime-avibactam (Table 2 and Fig. S1). Cefiderocol maintained activity against isolates resistant to the BL-BLI combinations, with a susceptibility of 95.1% against meropenem-vaborbactam-resistant isolates and 95.9% against imipenem-relebactam-resistant isolates. When tested against 37 ceftazidime-avibactam-resistant isolates, cefiderocol susceptibility was 89.2% (Table 2). Isolates resistant to one BL-BLI showed a higher resistance rate to other BL-BLIs. There were 23 isolates resistant to all 3 BL-BLI combinations, and susceptibility to cefiderocol was 91.3% (Table 2).

**TABLE 2:** Antimicrobial activity of cefiderocol and comparator agents tested against 8,047 *Enterobacterales* isolates^*d*^

Organism (no.)/antimicrobial agent	MIC (mg/L)			CLSI (%)^*a*^		
	MIC_50_	MIC_90_	MIC range	S	I	R
*Enterobacterales* (8,047)						
Cefiderocol	0.06	0.5	≤0.004 to >64	99.8	0.1	<0.1
Imipenem-relebactam	0.12	0.5	≤0.03 to >8	94.8^*b*^	0.3	0.7
Meropenem-vaborbactam	0.03	0.06	≤0.015 to >8	99.4	0.1	0.5
Ceftazidime-avibactam	0.12	0.25	≤0.015 to >32	99.5		0.5
Piperacillin-tazobactam	2	32	≤0.06 to >128	89	4.3	6.7
Meropenem	0.03	0.06	≤0.015 to >32	97.8	0.3	1.9
Colistin	0.25	>8	≤0.06 to >8	—^*c*^	83.6	16.4
CRE (169)						
Cefiderocol	0.5	4	0.008 to 8	98.2	1.8	0.0
Imipenem-relebactam	0.25	>8	0.06 to >8	63.9^*b*^	7.1	28.6
Meropenem-vaborbactam	1	>8	≤0.015 to >8	71.0	4.7	24.3
Ceftazidime-avibactam	1	>32	≤0.015 to >32	81.7		18.3
Piperacillin-tazobactam	>128	>128	2 to >128	0.6	3.6	95.9
Meropenem	16	>32	0.5 to >32	4.1	5.9	89.9
Colistin	0.25	>8	0.12 to >8	—^*c*^	78.7	21.3
Meropenem-vaborbactam resistant (41)						
Cefiderocol	1	4	0.03 to 8	95.1	4.9	0.0
Imipenem-relebactam	8	>8	0.5 to >8	2.4^*b*^	5.0	92.5
Meropenem-vaborbactam	>8	>8	>8 to >8	0.0	0.0	100
Ceftazidime-avibactam	>32	>32	0.25 to >32	43.9		56.1
Piperacillin-tazobactam	>128	>128	128 to >128	0.0	0.0	100
Meropenem	32	>32	8 to >32	0.0	0.0	100
Colistin	8	>8	0.12 to >8	—^*c*^	48.8	51.2
Imipenem-relebactam resistant (49)						
Cefiderocol	1	4	0.03 to 8	95.9	4.1	0.0
Imipenem-relebactam	8	>8	4 to >8	0.0^*b*^	0.0	100.0
Meropenem-vaborbactam	>8	>8	0.03 to >8	16.3	8.2	75.5
Ceftazidime-avibactam	>32	>32	0.12 to >32	40.8		59.2
Piperacillin-tazobactam	>128	>128	2 to >128	4.1	0.0	95.9
Meropenem	32	>32	0.06 to >32	6.1	4.1	89.8
Colistin	0.5	>8	0.12 to >8	—^*c*^	55.1	44.9
Ceftazidime-avibactam resistant (37)						
Cefiderocol	2	8	0.06 to >64	89.2	5.4	5.4
Imipenem-relebactam	>8	>8	0.25 to >8	5.4	2.7	91.9
Meropenem-vaborbactam	>8	>8	0.03 to >8	29.7	8.1	62.2
Ceftazidime-avibactam	>32	>32	16 to >32	0.0		100.0
Piperacillin-tazobactam	>128	>128	16 to >128	2.7	5.4	91.9
Meropenem	32	>32	0.12 to >32	21.6	0.0	78.4
Colistin	0.5	>8	0.12 to >8	—^*c*^	56.8	43.2
BL-BLI resistant (23)						
Cefiderocol	4	4	0.5 to 8	91.3	8.7	0.0
Imipenem-relebactam	>8	>8	8 to >8	0.0^*b*^	0.0	100.0
Meropenem-vaborbactam	>8	>8	>8 to >8	0.0	0.0	100.0
Ceftazidime-avibactam	>32	>32	>32 to >32	0.0		100.0
Piperacillin-tazobactam	>128	>128	128 to >128	0.0	0.0	100.0
Meropenem	>32	>32	16 to >32	0.0	0.0	100.0
Colistin	8	>8	0.12 to >8	—^*c*^	47.8	52.2

a Criteria as published by CLSI (2021).

b CLSI/FDA breakpoints were applied to all species but were approved for *Enterobacterales* except *Morganella*, Proteus, and *Providencia*.

c As CLSI removed the susceptible breakpoint for colistin, all wild-type isolates are considered intermediate.

d MIC_50_, MIC to inhibit growth of 50% of isolates; MIC_90_, MIC to inhibit growth of 90% of isolates; S, susceptible; I, intermediate; R, resistant.

### P. aeruginosa.

**FIG 3:**
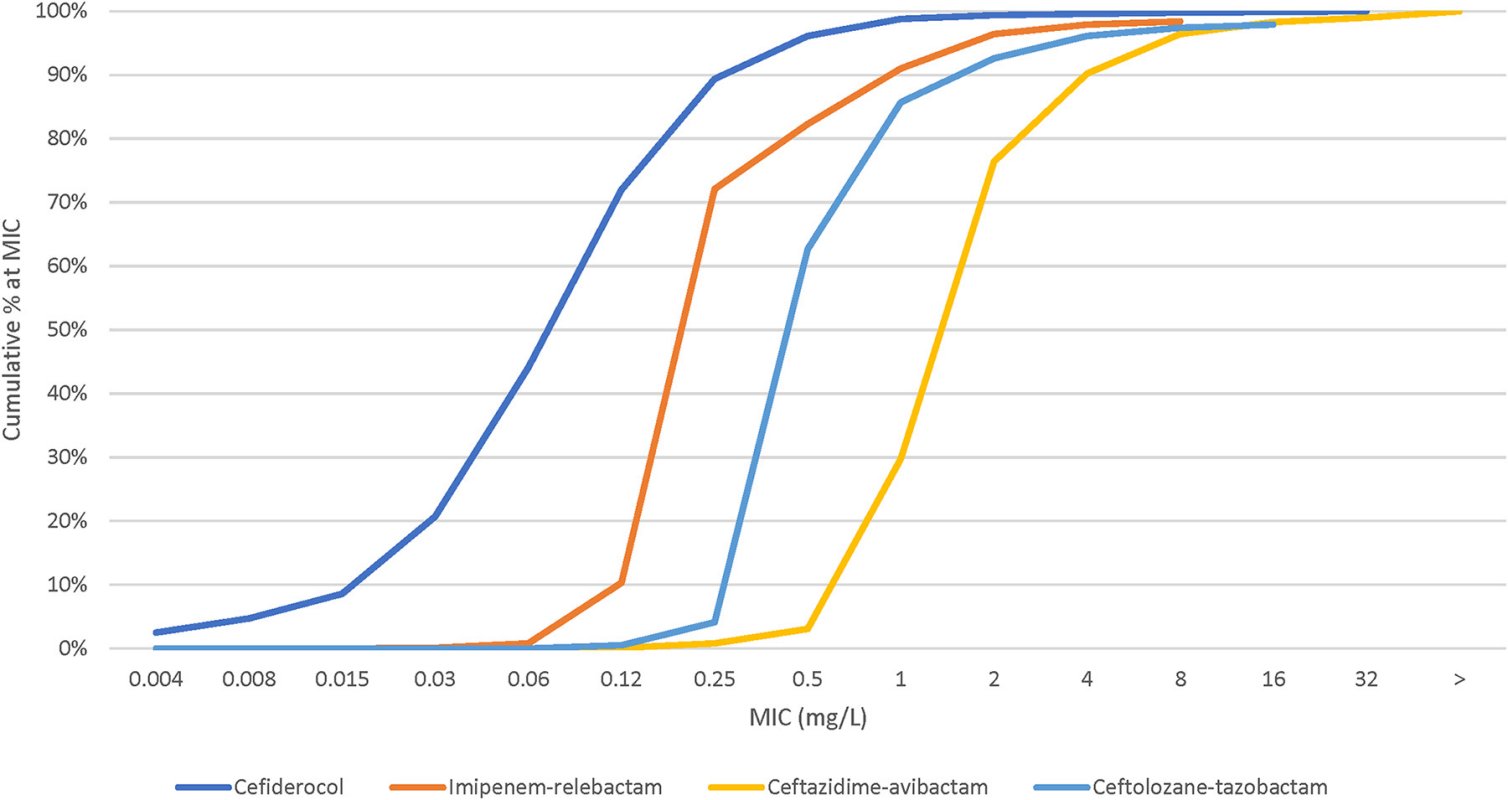
Cumulative percent MIC distribution of cefiderocol and comparators against P. aeruginosa isolates (*n *= 2,282).

Cefiderocol was the most active antimicrobial with MIC_50/90_ values of 0.12/0.5 mg/L (99.6% susceptible, CLSI) against all P. aeruginosa isolates (Table 3 and Fig. 3). Susceptibility to the tested agents for all P. aeruginosa isolates was ≥96% except for meropenem (78.1%) and piperacillin-tazobactam (78.0%). Susceptibility of XDR isolates to cefiderocol (MIC_50/90_, 0.12/1 mg/L) was 97.3% (Table 3; see also Fig. S2). Susceptibilities of XDR isolates to the 3 newer BL-BLI combinations—imipenem-relebactam, ceftazidime-avibactam, and ceftolozane-tazobactam—were lower than those to cefiderocol, at 73.0%, 73.4%, and 72.3%, respectively. Meropenem and piperacillin-tazobactam had poor activity against XDR isolates with 7.4% and 3.9% susceptibility rates, respectively.

**TABLE 3:** Antimicrobial activity of cefiderocol and comparator agents tested against 2,282 Pseudomonas aeruginosa isolates

Organism (no.)/antimicrobial agent	MIC (mg/L)			CLSI (%)^*a*^		
	MIC_50_	MIC_90_	MIC range	S	I	R
P. aeruginosa (2,282)						
Cefiderocol	0.12	0.5	≤0.004 to 32	99.6	0.2	0.2
Imipenem-relebactam	0.25	1	≤0.03 to >8	96.4	1.5	2.1
Ceftazidime-avibactam	2	4	0.06 to >32	96.4		3.6
Ceftolozane-tazobactam	0.5	2	≤0.12 to >16	96.1	1.3	2.6
Piperacillin-tazobactam	4	128	≤0.06 to >128	78	10.7	11.3
Meropenem	0.5	8	≤0.015 to >32	78.1	5.7	16.3
Colistin	1	1	≤0.06 to >8	—^*b*^	99.6	0.4
XDR (256)						
Cefiderocol	0.12	1	≤0.004 to 16	97.3	1.6	1.2
Imipenem-relebactam	2	>8	0.12 to >8	73.0	10.5	16.4
Ceftazidime-avibactam	8	32	0.5 to >32	73.4		26.6
Ceftolozane-tazobactam	2	>16	0.5 to >16	72.3	7.4	20.3
Piperacillin-tazobactam	128	>128	1 to >128	3.9	41.0	55.1
Meropenem	16	>32	0.25 to >32	7.4	12.5	80.1
Colistin	1	1	0.12 to >8	—^*b*^	99.2	0.8
Imipenem-relebactam resistant (48)						
Cefiderocol	0.12	1	0.015 to 2	100.0	0.0	0.0
Imipenem-relebactam	>8	>8	8 to >8	0.0	0.0	100.0
Ceftazidime-avibactam	16	>32	2 to >32	35.4		64.6
Ceftolozane-tazobactam	>16	>16	1 to >16	20.8	16.7	62.5
Piperacillin-tazobactam	64	>128	4 to >128	6.2	52.1	41.7
Meropenem	>32	>32	2 to >32	2.1	2.1	95.8
Colistin	1	1	0.25 to 2	—^*b*^	100.0	0.0
Ceftolozane-tazobactam resistant (60)						
Cefiderocol	0.25	8	0.015 to 32	88.3	5.0	6.7
Imipenem-relebactam	4	>8	0.25 to >8	43.3	6.7	50.0
Ceftazidime-avibactam	32	>32	2 to >32	25.0		75.0
Ceftolozane-tazobactam	>16	>16	16 to >16	0.0	0.0	100.0
Piperacillin-tazobactam	64	>128	4 to >128	6.7	46.7	46.7
Meropenem	16	>32	0.5 to >32	3.3	13.3	83.3
Colistin	1	1	0.12 to 2	—^*b*^	100.0	0.0
Ceftazidime-avibactam resistant (83)						
Cefiderocol	0.25	4	0.008 to 32	91.6	3.6	4.8
Imipenem-relebactam	4	>8	0.12 to >8	47	15.7	37.3
Ceftazidime-avibactam	16	>32	16 to >32	0.0		100.0
Ceftolozane-tazobactam	16	>16	1 to >16	37.3	8.4	54.2
Piperacillin-tazobactam	128	>128	4 to >128	3.6	34.9	61.4
Meropenem	32	>32	0.5 to >32	8.4	13.3	78.3
Colistin	1	1	0.12 to 2	—^*b*^	100.0	0.0
BL-BLI resistant (27)						
Cefiderocol	0.12	2	0.015 to 2	100.0	0.0	0.0
Imipenem-relebactam	>8	>8	8 to >8	0.0	0.0	100.0
Ceftazidime-avibactam	32	>32	16 to >32	0.0		100.0
Ceftolozane-tazobactam	>16	>16	>16 to >16	0.0	0.0	100.0
Piperacillin-tazobactam	64	>128	32 to >128	0.0	59.3	40.7
Meropenem	>32	>32	4 to >32	0.0	3.7	96.3
Colistin	1	1	0.5 to 2	—^*b*^	100.0	0.0

a Criteria as published by CLSI (2021).

b As CLSI removed the susceptible breakpoint for colistin, all wild-type isolates are considered intermediate.

Cefiderocol was a potent inhibitor of BL-BLI-resistant P. aeruginosa, with MIC_50_ values from 0.12 to 0.25 mg/L and MIC_90_ values from 1 to 8 mg/L (Table 3). Susceptibility to cefiderocol was the highest for 48 imipenem-relebactam-resistant isolates (100.0%, CLSI) and was slightly lower for 83 ceftazidime-avibactam-resistant isolates (91.6%) and 60 ceftolozane-tazobactam-resistant isolates (88.3%). Isolates that were resistant to each of the BL-BLI combinations were frequently resistant to the other BL-BLI combinations as well as other antimicrobials tested. Twenty-seven isolates were resistant to all 3 BL-BLI combinations and were 100.0% susceptible to cefiderocol (Table 3). Only colistin demonstrated susceptibility to all isolates per EUCAST criteria (Table S3). CLSI removed the susceptible category for colistin, classifying all wild-type isolates as intermediate.

### Acinetobacter and *Stenotrophomonas* spp.

**FIG 4:**
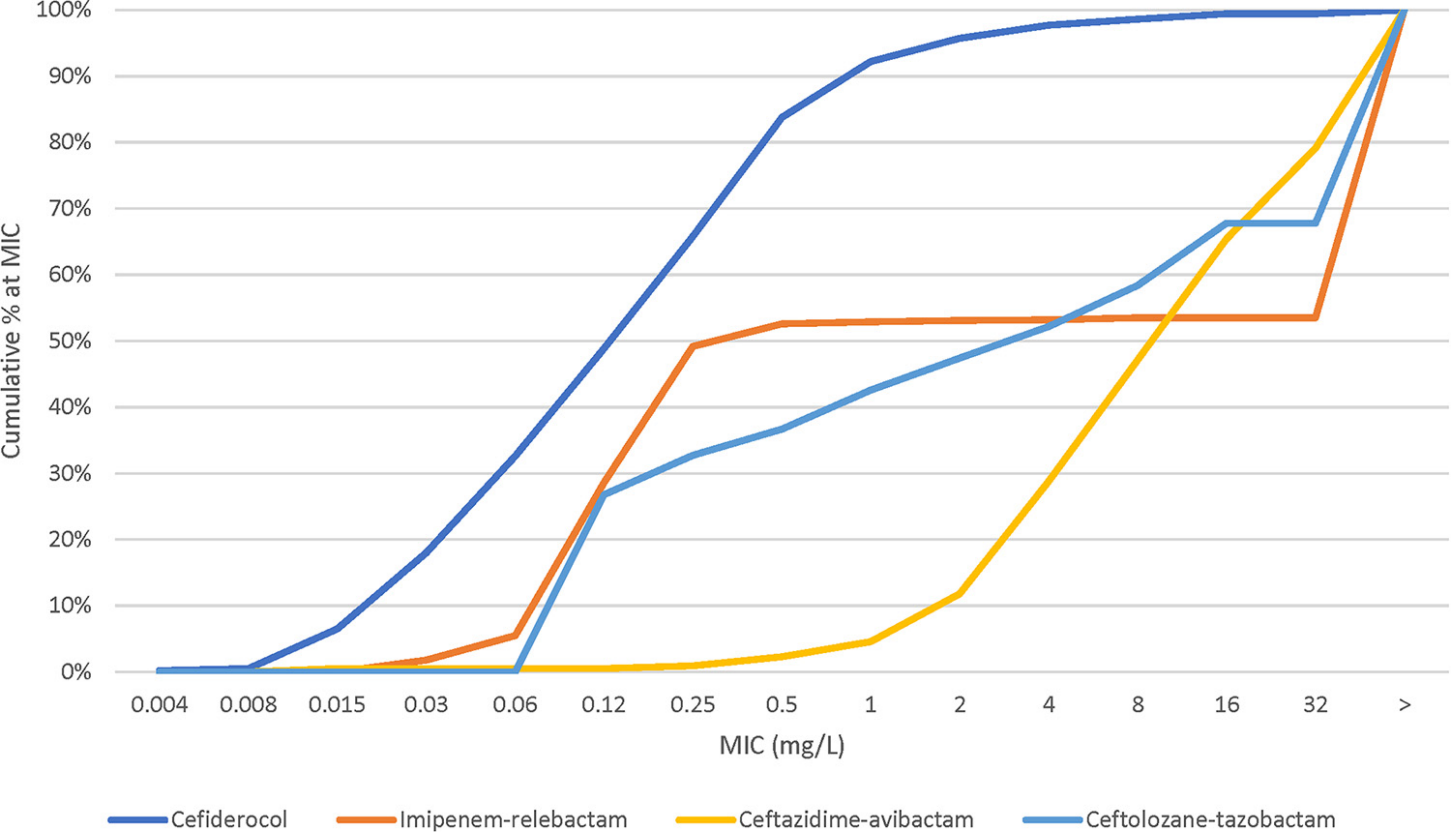
Cumulative percent MIC distribution of cefiderocol and comparators against Acinetobacter isolates (*n *= 650).

When tested against Acinetobacter spp. (650 isolates, including 586 of A. baumannii*-calcoaceticus* complex), susceptibility to cefiderocol was 97.7% (CLSI) (Table 4 and Fig. 4). Susceptibility to meropenem was 52.6% and susceptibility to imipenem-relebactam was 53.1% according to FDA breakpoints (Table S4). Susceptibility of the meropenem-resistant isolates to cefiderocol was 95.8% (Table 4 and Fig. S3). Susceptibilities to comparators were less than 9%, except for colistin, which was 76.4% susceptible by EUCAST criteria and 76.4% intermediate by CLSI criteria.

**TABLE 4:** Antimicrobial activity of cefiderocol and comparator agents tested against 650 Acinetobacter isolates

Organism (no.)/antimicrobial agent	MIC (mg/L)			CLSI (%)^*a*^		
	MIC_50_	MIC_90_	MIC range	S	I	R
Acinetobacter spp. (650)^*d*^						
Cefiderocol	0.25	1	≤0.004 to >64	97.7	0.9	1.4
Imipenem-relebactam	0.5	>8	≤0.03 to >8	53.1^*b*^	0.2	46.8
Ceftazidime	8	>32	0.25 to >32	50.8	4.5	44.8
Piperacillin-tazobactam	128	>128	≤0.06 to >128	45.8	2.2	52
Meropenem	1	>32	0.03 to >32	52.6	0.3	47.1
Ciprofloxacin	2	>4	≤0.008 to >4	49.1	1.1	49.8
Colistin	0.5	8	≤0.06 to >8	—^*c*^	86.3	13.7
Meropenem resistant (306)						
Cefiderocol	0.5	2	0.015 to >64	95.8	1.3	2.9
Imipenem-relebactam	>8	>8	0.25 to >8	0.3^*b*^	0.3	99.3
Ceftazidime	>32	>32	2 to >32	8.8	2.9	88.2
Piperacillin-tazobactam	>128	>128	≤0.06 to >128	1.0	0.3	98.7
Meropenem	>32	>32	8 to >32	0.0	0.0	100.0
Ciprofloxacin	>4	>4	1 to >4	0.7	0.3	99.0
Colistin	0.5	>8	0.12 to >8	—^*c*^	76.4	23.6

a Criteria as published by CLSI (2021).

b FDA criteria are shown, no CLSI breakpoints.

c As CLSI removed the susceptible breakpoint for colistin, all wild-type isolates are considered intermediate.

d Organisms include the following (no. of isolates): Acinetobacter baumannii (1), A. baumannii*-calcoaceticus* species complex (586), A. bereziniae (5), A. calcoaceticus (1), A. courvalinii (2), A. dispersus (1), A. guillouiae (1), A. gyllenbergii (1), A. johnsonii (4), A. junii (9), A. lwoffii (3), A. proteolyticus (1), A. radioresistens (14), A. schindleri (2), A. soli (1), A. ursingii (14), A. variabilis (1), A. vivianii (2), and Acinetobacter not identified to species level (1).

Stenotrophomonas maltophilia (*n *= 338) susceptibility to cefiderocol was 100.0% using CLSI 2021 breakpoints and 97.7% with CLSI 2022 breakpoints (Table 5). Other active agents were levofloxacin (82.5% susceptible), minocycline (99.4%), and trimethoprim-sulfamethoxazole (97.9%).

**TABLE 5:** Antimicrobial activity of cefiderocol and comparator agents tested against 338 Stenotrophomonas maltophilia isolates

Antimicrobial agent against S. maltophilia (*n* = 338)	MIC (mg/L)			CLSI (%)^*a*^		
	MIC_50_	MIC_90_	MIC range	S	I	R
Cefiderocol	0.12	0.5	0.015 to 4	100.0, 97.9^*b*^	0.0	0.0
Ceftazidime	>32	>32	1 to >32	16.6	11.6	71.8
Levofloxacin	1	8	0.12 to 32	82.5	7.4	10.1
Trimethoprim-sulfamethoxazole	≤0.12	0.5	≤0.12 to >4	97.9		2.1
Minocycline	0.5	1	0.12 to 8	99.4	0.6	0.0
Colistin	8	>8	0.12 to >8			

a Criteria as published by CLSI (2021).

b CLSI 2021 (≤4/8/≥16 mg/L) and 2022 (≤1/−/− mg/L) breakpoints shown.

### Comparison of activities against the main organism groups from the United States and Europe.

The activities of cefiderocol against U.S. and European isolates were very similar. U.S. and European cefiderocol MIC_50/90_ values for *Enterobacterales* isolates were 0.06 to 0.12/0.5 mg/L (Table 6). *Enterobacterales* susceptibilities to cefiderocol were 99.8% (CLSI) for the U.S. and European isolates. Piperacillin-tazobactam and meropenem were less active in Europe than in the United States. Susceptibilities to piperacillin-tazobactam were 91.4% for the United States and 86.6% for Europe. Susceptibilities to meropenem were 99.1% for the United States and 96.6% for Europe. The CRE rate was 0.9% in the United States and 3.3% in Europe.

**TABLE 6:** Comparison of susceptibilities to cefiderocol and comparators between the United States and Europe

Organism/antimicrobial agent	MIC (mg/L)		CLSI,^*a*^ % S	MIC (mg/L)		CLSI,^*a*^ % S
	MIC_50_	MIC_90_		MIC_50_	MIC_90_	
*Enterobacterales*	USA, *n* = 4,053			Europe, *n* = 3,994		
Cefiderocol	0.06	0.5	99.8	0.12	0.5	99.8
Imipenem-relebactam	0.12	0.5	95.6^*b*^	0.12	1	94.0^*b*^
Meropenem-vaborbactam	0.03	0.06	99.9	0.03	0.06	98.9
Ceftazidime-avibactam	0.12	0.25	>99.9	0.12	0.5	99.1
Piperacillin-tazobactam	2	16	91.4	2.0	64.0	86.6
Meropenem	0.03	0.06	99.1	0.03	0.06	96.6
Colistin	0.25	>8	83.3^*c*^	0.25	>8	83.9^*c*^
P. aeruginosa	USA, *n* = 1,069			Europe, *n* = 1,213		
Cefiderocol	0.12	0.5	99.5	0.12	0.5	99.7
Imipenem-relebactam	0.25	1	97.3	0.25	1	95.5
Ceftazidime-avibactam	2	8	96.4	2	4	96.4
Ceftolozane-tazobactam	0.5	2	97.8	0.5	2	94.6
Piperacillin-tazobactam	4	128	79.2	4	128	76.9
Meropenem	0.5	8	79	0.5	8	77.3
Colistin	1	1	99.6^*c*^	1	1	99.7^*c*^
A. baumannii*-calcoaceticus* complex	USA, *n* = 248			Europe, *n* = 340		
Cefiderocol	0.25	1	97.6	0.25	1	97.4
Imipenem-relebactam	0.25	>8	62.9^*d*^	>8	>8	62.9^*d*^
Ceftazidime	8	>32	60.9	>32	>32	37.6
Piperacillin-tazobactam	16	>128	51.2	>128	>128	33.3
Meropenem	1	>32	61.7	>32	>32	37.6
Ciprofloxacin	1	>4	54.8	>4	>4	37.1
Colistin	0.5	1	93.9^*c*^	0.5	>8	80.9

a Criteria as published by CLSI (2021).

b *Enterobacterales* breakpoints were applied to all organisms including *Morganellaceae*, which are intrinsically less susceptible.

c As there is no susceptible CLSI breakpoint, the intermediate category is shown.

d FDA breakpoints shown; no CLSI breakpoints.

The percentage of U.S. and European P. aeruginosa isolates susceptible to cefiderocol (MIC_50/90_, 0.12/0.5 mg/L) was 99.5% and 99.7% (CLSI), respectively (Table 6). Susceptibility to meropenem was 79.0% in the United States and 77.3% in Europe. Susceptibility to piperacillin-tazobactam was 79.2% in the United States and 76.9% in Europe.

Cefiderocol activities against A. baumannii*-calcoaceticus* complex were also similar in the United States and Europe, with MIC_50/90_ values of 0.25/1 mg/L for both regions (Table 6). Susceptibilities to cefiderocol were 97.6/97.4% in the United States and Europe, respectively. In contrast, susceptibility to meropenem was lower in Europe, with 37.6% compared to 61.7% in the United States.

## DISCUSSION

Cefiderocol is the first siderophore-linked cephalosporin approved for use. In our study, this novel drug had a broad spectrum of activity against a large 2020 collection of Gram-negative isolates. Cefiderocol was very active against *Enterobacterales* including CRE and XDR isolates. Most importantly, cefiderocol retained good activity against isolates resistant to the recently approved BL-BLI combinations. These BL-BLI-resistant isolates are challenging to treat due to very limited therapeutic options. Cefiderocol also had potent activity against nonfermentative, Gram-negative organisms, including XDR and BL-BLI-resistant P. aeruginosa, and against meropenem-resistant Acinetobacter spp. Isolates resistant to cefiderocol were observed but rare, representing <1.5% overall of *Enterobacterales*, P. aeruginosa, Acinetobacter species, and S. maltophilia isolates in this study using CLSI breakpoints.

The susceptibility to cefiderocol of *Enterobacterales* isolates resistant to one or more BL-BLIs observed in this study has been noted by others. This susceptibility may be related to the increased stability of cefiderocol to hydrolysis by the enzymes responsible for resistance to the BL-BLIs, which include OXA-48-like carbapenemases and metallo-β-lactamases as well as porin defects or loss (12, 13). We observed that P. aeruginosa isolates resistant to one BL-BLI combination were frequently resistant to other BL-BLIs but susceptible to cefiderocol, including 27 isolates that were coresistant to all 3 BL-BLI combinations. Possible BL-BLI resistance mechanisms for these isolates are overexpression of PDC (Pseudomonas-derived cephalosporinase) and/or MexAB or MexXY efflux, as well as oprD loss (14). Other studies have observed that cefiderocol MIC values of *Enterobacterales* or P. aeruginosa were not correlated with efflux increases or porin defects, suggesting that cefiderocol entry via the iron-transport system may bypass porin changes and that the drug is a poor substrate for efflux pumps (13, 15). Cefiderocol is also more resistant to hydrolysis by chromosomal cephalosporinases (8, 9).

For Acinetobacter, cefiderocol resistance was <1.5% when applying CLSI breakpoints. There are a limited number of antimicrobials with indications and breakpoints for Acinetobacter spp. that have useful activity, particularly against carbapenem-resistant Acinetobacter. The excellent *in vitro* susceptibility of meropenem-resistant Acinetobacter spp. to cefiderocol (95.8%, CLSI) suggests that this drug may be an important treatment alternative to colistin, which had a resistance rate of 23.6%. It should be noted that CLSI removed the susceptible category for colistin due to its toxicity and poor efficacy when used systemically to treat pneumonia (16).

One limitation of our study is that there was no molecular characterization of the antimicrobial-resistant isolates. However, these mechanisms will be investigated and described in future publications. A second limitation is that there was no patient chart review. Therefore, no patient treatment or outcome information is available, including whether there was any cefiderocol use in the institutions that submitted isolates.

These *in vitro* data suggest that cefiderocol may be an important therapeutic option for the treatment of infections caused by Gram-negative organisms, including isolates resistant to carbapenems and BL-BLI combinations, which have limited treatment options. Although resistance to cefiderocol remains very uncommon, there is a need to continue antimicrobial surveillance.

## MATERIALS AND METHODS

A total of 8,047 *Enterobacterales*, 2,282 P. aeruginosa, 650 Acinetobacter species including 588 A. baumannii*-calcoaceticus* complex, and 338 S. maltophilia isolates were consecutively collected from patients in 66 hospitals located in the United States and Europe during 2020 according to a common protocol as previously described (17). A list of the number of isolates by country is shown in Table S1 in the supplemental material. Isolates from all infection types were included in this analysis.

Susceptibility testing was performed using the Clinical and Laboratory Standards Institute (CLSI) broth microdilution method (18). Cefiderocol was tested in iron-depleted, cation-adjusted Mueller-Hinton broth that was prepared according to CLSI guidelines. All CLSI and European Committee on Antimicrobial Susceptibility Testing (EUCAST) quality control (QC) strains were within established ranges (18, 19).

Breakpoints applied to cefiderocol are shown in Table 1 (18, 20, 21).

CLSI, FDA, and EUCAST breakpoints were used for comparator antimicrobials as available. CLSI susceptibilities for all antimicrobials are shown in Tables 2 to 6; EUCAST and FDA susceptibilities are shown in Tables S2 to S6 in the supplemental material.

The *Enterobacterales* and P. aeruginosa breakpoints for several antimicrobial agents were recently changed by EUCAST to recategorize all isolates in the wild-type population as “susceptible, increased exposure (intermediate)” (20). The arbitrary susceptible breakpoint of ≤0.001 mg/L was chosen by EUCAST to ensure that no isolates were labeled susceptible to these agents. As a result, P. aeruginosa isolates that were considered to be susceptible to piperacillin-tazobactam, cefepime, ceftazidime, imipenem, aztreonam, and ciprofloxacin, as well as Proteus spp., *Providencia* spp., and Morganella morganii isolates that were considered susceptible to imipenem, now are shown as “intermediate” in this study. CLSI also recently removed the susceptible category for colistin, reporting only intermediate or resistant categories for *Enterobacterales* and P. aeruginosa (18).

Carbapenem resistance was identified by applying CLSI breakpoints, as isolates having an MIC of >2 mg/L to meropenem and/or imipenem (18). An imipenem MIC was not applied to *Morganella*, Proteus, or *Providencia* spp. Extensive drug resistance was defined as isolates susceptible to ≤2 of the following drug classes: antipseudomonal cephalosporins, antipseudomonal BL-BLIs, antipseudomonal fluoroquinolones, aminoglycosides, carbapenems, and polymyxins (22). Agents in these classes were tested for resistance phenotype determination; not all data are shown. Isolates were not genetically characterized for resistance mechanisms.

Other antimicrobials tested included the BL-BLI combinations ceftazidime-avibactam, ceftolozane-tazobactam, imipenem-relebactam, and meropenem-vaborbactam. This study also analyzed isolate subgroups resistant to these combinations based on CLSI breakpoints. All combination agents were tested with a fixed 4 mg/L of inhibitor, except for meropenem-vaborbactam, which was tested with a fixed 8 mg/L of vaborbactam per CLSI and EUCAST criteria (18, 20).
