# Supplementary material for: In Vitro Activity of Cefiderocol against U.S. and European Gram-Negative Clinical Isolates Collected in 2020 as Part of the SENTRY Antimicrobial Surveillance Program
Source: Microbiol Spectr. 2022 Mar 9;10(2):e02712-21. doi: 10.1128/spectrum.02712-21 (PMC9045385; doi:10.1128/spectrum.02712-21)

Supplemental Material

Table 1 Number of isolates and sites by country

Country	Number of sites	Number of isolates
Belgium	1	142
Czech Republic	1	121
France	3	432
Germany	6	1049
Greece	1	257
Hungary	1	135
Ireland	1	199
Israel	2	171
Italy	4	810
Poland	1	192
Portugal	1	146
Romania	1	80
Slovenia	1	202
Spain	3	604
Sweden	2	235
Switzerland	1	204
Turkey	2	395
UK	3	357
USA	31	5702

Table 2 Antimicrobial activity of cefiderocol and comparator agents tested against 8,047 Enterobacterales

Organism/ Antimicrobial agent	mg/L			EUCAST[a]			FDA[a]		
	MIC_{50}	MIC_{90}	MIC range	%S	%I	%R	%S	%I	%R
**Enterobacterales (n=8,047)**									
Cefiderocol	0.06	0.5	≤0.004 to >64	99.1		0.9	99.8	0.1	<0.1
Imipenem-relebactam	0.12	0.5	≤0.03 to >8	98.9 [b]		1.1	94.8	4	1.1
Meropenem-vaborbactam	0.03	0.06	≤0.015 to >8	99.5		0.5	99.4	0.1	0.5
Ceftazidime-avibactam	0.12	0.25	≤0.015 to >32	99.5		0.5	99.5		0.5
Piperacillin-tazobactam	2	32	≤0.06 to >128	84.9		15.1	89.0	4.3	6.7
Meropenem	0.03	0.06	≤0.015 to >32	98.1	0.5	1.4	97.8	0.3	1.9
Colistin	0.25	>8	≤0.06 to >8	83.6		16.4			
CRE (n=169)									
Cefiderocol	0.5	4	0.008 to 8	87.6		12.4	98.2	1.8	0
Imipenem-relebactam	0.25	>8	0.06 to >8	71 [b]		29	63.9	7.1	29
Meropenem-vaborbactam	1	>8	≤0.015 to >8	75.7		24.3	71.0	4.7	24.3
Ceftazidime-avibactam	1	>32	≤0.015 to >32	81.7		18.3	81.7		18.3
Piperacillin-tazobactam	>128	>128	2 to >128	0.6		99.4	0.6	3.6	95.9
Meropenem	16	>32	0.5 to >32	10.1	23.1	66.9	4.1	5.9	89.9
Colistin	0.25	>8	0.12 to >8	78.7		21.3			
Meropenem-vaborbactam-resistant (n=41)									
Cefiderocol	1	4	0.03 to 8	70.7		29.3	95.1	4.9	0
Imipenem-relebactam	8	>8	0.5 to >8	7.3 [b]		92.7	2.4	4.9	92.7
Meropenem-vaborbactam	>8	>8	>8 to >8	0.0		100	0.0	0.0	100.0
Ceftazidime-avibactam	>32	>32	0.25 to >32	43.9		56.1	43.9		56.1
Piperacillin-tazobactam	>128	>128	128 to >128	0.0		100	0.0	0.0	100
Meropenem	32	>32	8 to >32	0.0	2.4	97.6	0.0	0.0	100
Colistin	8	>8	0.12 to >8	48.8		51.2			
Imipenem-relebactam-resistant (n=49)									
Cefiderocol	1	4	0.03 to 8	69.4		30.6	95.9 [b]	4.1	0
Imipenem-relebactam	8	>8	4 to >8	0 [b]		100	0.0	0.0	100
Meropenem-vaborbactam	>8	>8	0.03 to >8	24.5		75.5	16.3	8.2	75.5
Ceftazidime-avibactam	>32	>32	0.12 to >32	40.8		59.2	40.8		59.2
Piperacillin-tazobactam	>128	>128	2 to >128	4.1		95.9	4.1	0	95.9
Meropenem	32	>32	0.06 to >32	10.2	10.2	79.6	6.1	4.1	89.8
Colistin	0.5	>8	0.12 to >8	55.1		44.9			
Ceftazidime-avibactam-resistant (n=37)									

Organism/ Antimicrobial agent	mg/L			EUCAST[a]			FDA[a]		
	MIC$_{50}$	MIC$_{90}$	MIC range	%S	%I	%R	%S	%I	%R
Cefiderocol	2	8	0.06 to >64	54.1		45.9	89.2	5.4	5.4
Imipenem-relebactam	>8	>8	0.25 to >8	8.1 [b]		91.9	5.4	2.7	91.9
Meropenem-vaborbactam	>8	>8	0.03 to >8	37.8		62.2	29.7	8.1	62.2
Ceftazidime-avibactam	>32	>32	16 to >32	0		100	0.0		100
Piperacillin-tazobactam	>128	>128	16 to >128	0		100	2.7	5.4	91.9
Meropenem	32	>32	0.12 to >32	21.6	13.5	64.9	21.6	0.0	78.4
Colistin	0.5	>8	0.12 to >8	56.8		43.2			
BL/BLI R (23)									
Cefiderocol	4	4	0.5 to 8	47.8		52.2	91.3	8.7	0.0
Imipenem-relebactam	>8	>8	8 to >8	0.0 [b]		100.0	0.0	0.0	100.0
Meropenem-vaborbactam	>8	>8	>8 to >8	0.0		100.0	0.0	0.0	100.0
Ceftazidime-avibactam	>32	>32	>32 to >32	0.0		100.0	0.0		100.0
Piperacillin-tazobactam	>128	>128	128 to >128	0.0		100.0	0.0	0.0	100.0
Meropenem	>32	>32	16 to >32	0.0		100.0	0.0	0.0	100.0
Colistin	8	>8	0.12 to >8	47.8		52.2			

[a] Criteria as published by EUCAST (2021), and FDA (2021).
[b] CLSI/FDA breakpoints were applied to all species but were approved for Enterobacterales except *Morganella*, *Proteus*, and *Providencia* and EUCAST excludes *Morganellaceae*.

MIC$_{50}$ mg/L, minimal inhibitory concentration to inhibit growth of 50% of isolates; MIC$_{90}$ mg/L, minimal inhibitory concentration to inhibit growth of 90% of isolates; S, susceptible, I, intermediate, R, resistant

Table 3 Antimicrobial activity of cefiderocol and comparator agents tested against 2,282 *Pseudomonas aeruginosa*

	mg/L			EUCAST[a]			FDA[a]		
**Organism/ Antimicrobial agent**	MIC$_{50}$	MIC$_{90}$	MIC range	%S	%I	%R	%S	%I	%R
***P. aeruginosa* (2,282)**									
Cefiderocol	0.12	0.5	≤0.004 to 32	99.4		0.6	98.8	0.6	0.6
Imipenem-relebactam	0.25	1	≤0.03 to >8	96.4		3.6	96.4	1.5	2.1
Ceftazidime-avibactam	2	4	0.06 to >32	96.4		3.6	96.4		3.6
Ceftolozane-tazobactam	0.5	2	≤0.12 to >16	96.1		3.9	96.1	1.3	2.6
Piperacillin-tazobactam	4	128	≤0.06 to >128	[b]	78	22	78.0	10.7	11.3
Meropenem	0.5	8	≤0.015 to >32	78.1	12.1	9.9	78.1	5.7	16.3
Colistin	1	1	≤0.06 to >8	99.6		0.4			
XDR (256)									
Cefiderocol	0.12	1	≤0.004 to 16	96.9		3.1	94.1	2.7	3.1
Imipenem-relebactam	2	>8	0.12 to >8	73		27	73.0	10.5	16.4
Ceftazidime-avibactam	8	32	0.5 to >32	73.4		26.6	73.4		26.6
Ceftolozane-tazobactam	2	>16	0.5 to >16	72.3		27.7	72.3	7.4	20.3
Piperacillin-tazobactam	128	>128	1 to >128	[b]	3.9	96.1	3.9	41	55.1
Meropenem	16	>32	0.25 to >32	7.4	34	58.6	7.4	12.5	80.1
Colistin	1	1	0.12 to >8	99.2		0.8			
Imipenem-relebactam-resistant (48)									
Cefiderocol	0.12	1	0.015 to 2	100		0	93.8	6.2	0
Imipenem-relebactam	>8	>8	8 to >8	0		100	0.0	0.0	100
Ceftazidime-avibactam	16	>32	2 to >32	35.4		64.6	35.4		64.6
Ceftolozane-tazobactam	>16	>16	1 to >16	20.8		79.2	20.8	16.7	62.5
Piperacillin-tazobactam	64	>128	4 to >128	[b]	6.2	93.8	6.2	52.1	41.7
Meropenem	>32	>32	2 to >32	2.1	4.2	93.8	2.1	2.1	95.8
Colistin	1	1	0.25 to 2	100.0		0.0			
Ceftolozane-tazobactam-resistant (60)									
Cefiderocol	0.25	8	0.015 to 32	85.0		15	75.0	10	15
Imipenem-relebactam	4	>8	0.25 to >8	43.3		56.7	43.3	6.7	50
Ceftazidime-avibactam	32	>32	2 to >32	25.0		75	25.0		75
Ceftolozane-tazobactam	>16	>16	16 to >16	0.0		100.0	0.0	0.0	100.0
Piperacillin-tazobactam	64	>128	4 to >128	[b]	6.7	93.3	6.7	46.7	46.7
Meropenem	16	>32	0.5 to >32	3.3	28.3	68.3	3.3	13.3	83.3
Colistin	1	1	0.12 to 2	100.0		0.0			
Ceftazidime-avibactam-resistant (83)									

	mg/L			EUCAST[a]			FDA[a]		
**Organism/ Antimicrobial agent**	MIC_{50}	MIC_{90}	MIC range	%S	%I	%R	%S	%I	%R
Cefiderocol	0.25	4	0.008 to 32	89.2		10.8	83.1	6	10.8
Imipenem-relebactam	4	>8	0.12 to >8	47		53	47.0	15.7	37.3
Ceftazidime-avibactam	16	>32	16 to >32	0.0		100.0	0.0		10.00
Ceftolozane-tazobactam	16	>16	1 to >16	37.3		62.7	37.3	8.4	54.2
Piperacillin-tazobactam	128	>128	4 to >128	[b]	3.6	96.4	3.6	34.9	61.4
Meropenem	32	>32	0.5 to >32	8.4	21.7	69.9	8.4	13.3	78.3
Colistin	1	1	0.12 to 2	100.0		0.0			
BL/BLI-R (27)									
Cefiderocol	0.12	2	0.015 to 2	100.0		0.0	88.9	11.1	0.0
Imipenem-relebactam	>8	>8	8 to >8	0.0		100.0	0.0	0.0	100.0
Ceftazidime-avibactam	32	>32	16 to >32	0.0		100.0	0.0		100.0
Ceftolozane-tazobactam	>16	>16	>16 to >16	0.0		100.0	0.0	0.0	100.0
Piperacillin-tazobactam	64	>128	32 to >128	[b]	0.0	100.0	0.0	59.3	40.7
Meropenem	>32	>32	4 to >32	0.0		100.0	0.0	3.7	96.3
Colistin	1	1	0.5 to 2	100.0		0.0			

[a] Criteria as published by EUCAST (2021), and FDA (2021).
[b] An arbitrary susceptible breakpoint of ≤0.001 mg/L has been published by EUCAST indicating that susceptible should not be reported for this organism-agent combination and intermediate should be interpreted as susceptible increased exposure.

Table 4 Antimicrobial activity of cefiderocol and comparator agents tested against 650 *Acinetobacter* spp.

	mg/L			EUCAST[a]			FDA[a]		
**Antimicrobial agent**	**MIC_{50}**	**MIC_{90}**	**MIC range**	**%S**	**%I**	**%R**	**%S**	**%I**	**%R**
Acinetobacter spp. (650)									
Cefiderocol	0.25	1	≤0.004 to >64	95.7[b]		4.3	92.2	3.5	4.3
Imipenem-relebactam	0.5	>8	≤0.03 to >8	53.1		46.9	53.1	0.2	46.8
Ceftazidime	8	>32	0.25 to >32				50.8	4.5	44.8
Piperacillin-tazobactam	128	>128	≤0.06 to >128				45.8	2.2	52
Meropenem	1	>32	0.03 to >32	52.6	0.6	46.8	52.6	0.3	47.1
Ciprofloxacin	2	>4	≤0.008 to >4	[c]	49.1	50.9			
Colistin	0.5	8	≤0.06 to >8	86.3		13.7			
Meropenem-resistant (306)									
Cefiderocol	0.5	2	0.015 to >64	91.5[b]		8.5	85.6	5.9	8.5
Imipenem-relebactam	>8	>8	0.25 to >8	0.3		99.7	0.3	0.3	99.3
Ceftazidime	>32	>32	2 to >32				8.8	2.9	88.2
Piperacillin-tazobactam	>128	>128	≤0.06 to >128				1.0	0.3	98.7
Meropenem	>32	>32	8 to >32	0.0	0.7	99.3	0.0	0.0	100
Ciprofloxacin	>4	>4	1 to >4	[c]	0.7	99.3			
Colistin	0.5	>8	0.12 to >8	76.4		23.6			

[a] Criteria as published by EUCAST (2021), and FDA (2021).
[b] EUCAST non-species specific pk/pd breakpoint used.
[c] An arbitrary susceptible breakpoint of ≤0.001 mg/L has been published by EUCAST indicating that susceptible should not be reported for this organism-agent combination and intermediate should be interpreted as susceptible increased exposure.

Organisms include: *Acinetobacter baumannii* (1), *A. baumannii-calcoaceticus* species complex (586), *A. berezinae* (5), *A. calcoaceticus* (1), *A. courvalinii* (2), *A. dispersus* (1), *A. guillouiae* (1), *A. gyllenbergii* (1), *A. johnsonii* (4), *A. junii* (9), *A. lwoffii* (3), *A. proteolyticus* (1), *A. radioresistens* (14), *A. schindleri* (2), *A. soli* (1), *A. ursingii* (14), *A. variabilis* (1), *A. vivianii* (2), and unspeciated *Acinetobacter* (1).

Table 5 Antimicrobial activity of cefiderocol and comparator agents tested against 338 *Stenotrophomonas maltophilia*

Organism/ Antimicrobial agent	mg/L			EUCAST[a]			FDA[a]		
	MIC_{50}	MIC_{90}	MIC range	%S	%I	%R	%S	%I	%R
S. maltophilila **(n=338)**									
Cefiderocol	0.12	0.5	0.015 to 4	99.7[c]		0.3			
Ceftazidime	>32	>32	1 to >32				16.6	11.6	71.8
Levofloxacin	1	8	0.12 to 32						
Trimethoprim-sulfamethoxazole	≤0.12	0.5	≤0.12 to >4	[d]	98.2	1.8			
Minocycline	0.5	1	0.12 to 8						
Colistin	8	>8	0.12 to >8						

[a] Criteria as published by EUCAST (2021), and FDA (2021).
[b] CLSI 2021 (≤4/8≥16 mg/L) and 2022 (≤1/-/- mg/L) breakpoints shown.
[c] EUCAST non-species specific pk/pd breakpoint used.
[d] An arbitrary susceptible breakpoint of ≤0.001 mg/L has been published by EUCAST indicating that susceptible should not be reported for this organism-agent combination and intermediate should be interpreted as susceptible increased exposure.

Table 6 Comparison of susceptibilities to cefiderocol and comparators between the US and Europe

Antimicrobial agent	mg/L		EUCAST[a]	FDA[a]	mg/L		EUCAST[a]	FDA[a]
	MIC_{50}	MIC_{90}	%S	%S	MIC_{50}	MIC_{90}	%S	%S
Enterobacterales	US n=4,053				Europe n=3,994			
Cefiderocol	0.06	0.5	99.2	99.8	0.12	0.5	98.9	99.8
Imipenem-relebactam	0.12	0.5	99.5	95.6	0.12	1	98.2	94.0
Meropenem-vaborbactam	0.03	0.06	99.9	99.9	0.03	0.06	99.0	98.9
Ceftazidime-avibactam	0.12	0.25	>99.9	>99.9	0.12	0.5	99.1	99.1
Piperacillin-tazobactam	2	16	88.0	91.4	2.0	64.0	81.8	86.6
Meropenem	0.03	0.06	99.3 [c]	99.1	0.03	0.06	96.9 [c]	96.6
Colistin	0.25	>8	83.3		0.25	>8	83.9	
P. aeruginosa	US n=1,069				Europe n=1,213			
Cefiderocol	0.12	0.5	99.4	98.9	0.12	0.5	99.4	98.8
Imipenem-relebactam	0.25	1	97.3	97.3	0.25	1	95.5	95.5
Ceftazidime-avibactam	2	8	96.4	96.4	2	4	96.4	96.4
Ceftolozane-tazobactam	0.5	2	97.8	97.8	0.5	2	94.6	94.6
Piperacillin-tazobactam	4	128	79.2 [c]	79.2	4	128	76.9 [c]	76.9
Meropenem	0.5	8	79.0	79.0	0.5	8	77.3	77.3
Colistin	1	1	99.6		1	1	99.7	
A. baumannii-calcoacetius complex	US n=248				Europe n=340			
Cefiderocol	0.25	1	96.4	90.3	0.25	1	94.4	92.1
Imipenem-relebactam	0.25	>8	62.9	62.9	>8	>8	37.6	37.6
Ceftazidime	8	>32		60.9	>32	>32		37.6
Piperacillin-tazobactam	16	>128		51.2	>128	>128		33.3
Meropenem	1	>32	61.7	61.7	>32	>32	37.6	37.6
Ciprofloxacin	1	>4	54.8[c]		>4	>4	37.1 [c]	
Colistin	0.5	1	93.9		0.5	>8	80.9	

[a] Criteria as published by EUCAST (2021), and FDA (2021).
[b] Enterobacterales breakpoints were applied to all organisms including Morganellaceae, which are intrinsically less susceptible..
[c] As an arbitrary susceptible breakpoint of ≤0.001 mg/L has been published by EUCAST indicating that susceptible should not be reported for this organism-agent combination, susceptible-increased exposure is shown.

Supplemental Figure 1. Carbapenem-resistant Enterobacterales

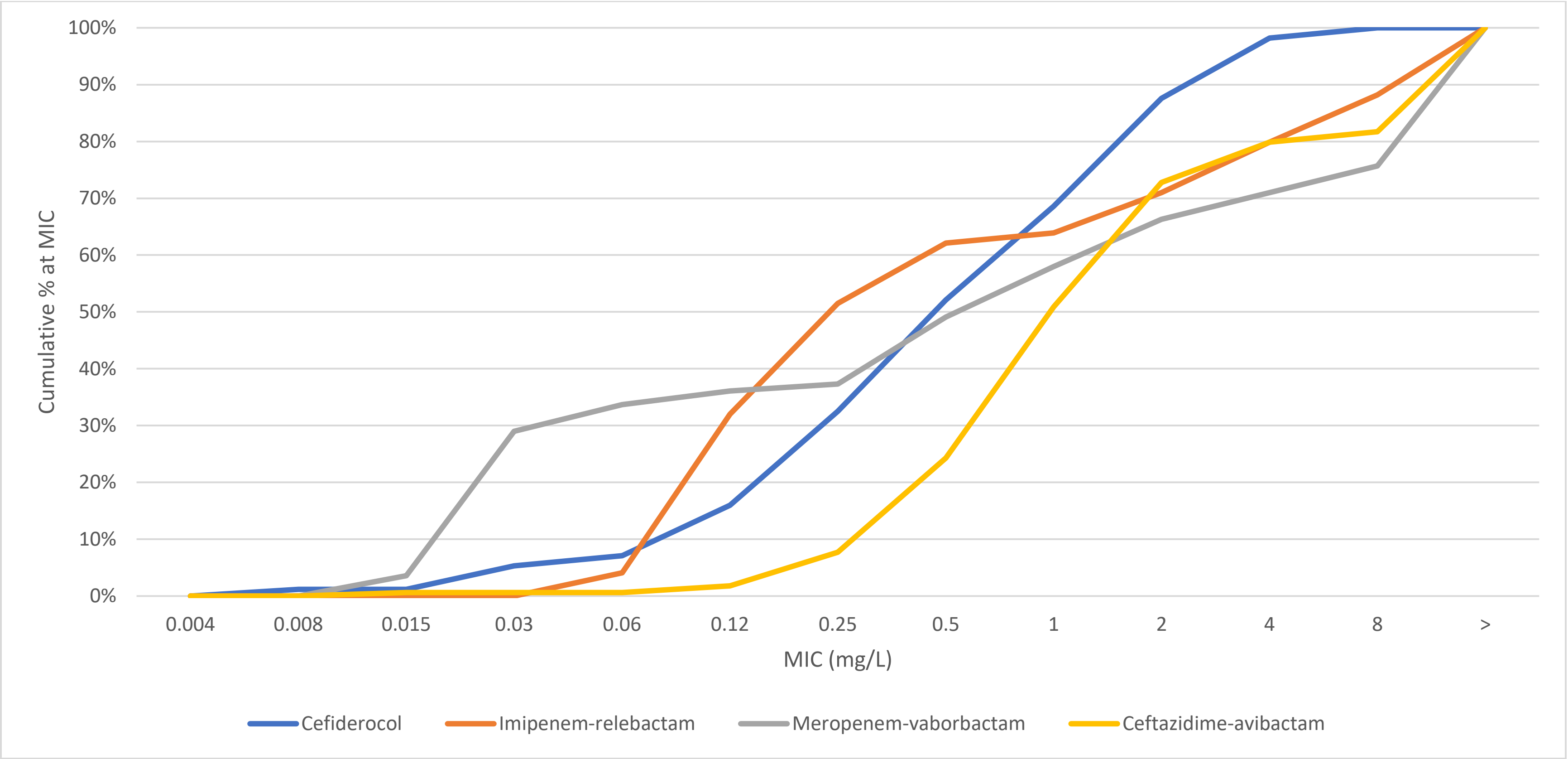

Supplemental figure 2. XDR *P. aeruginosa*

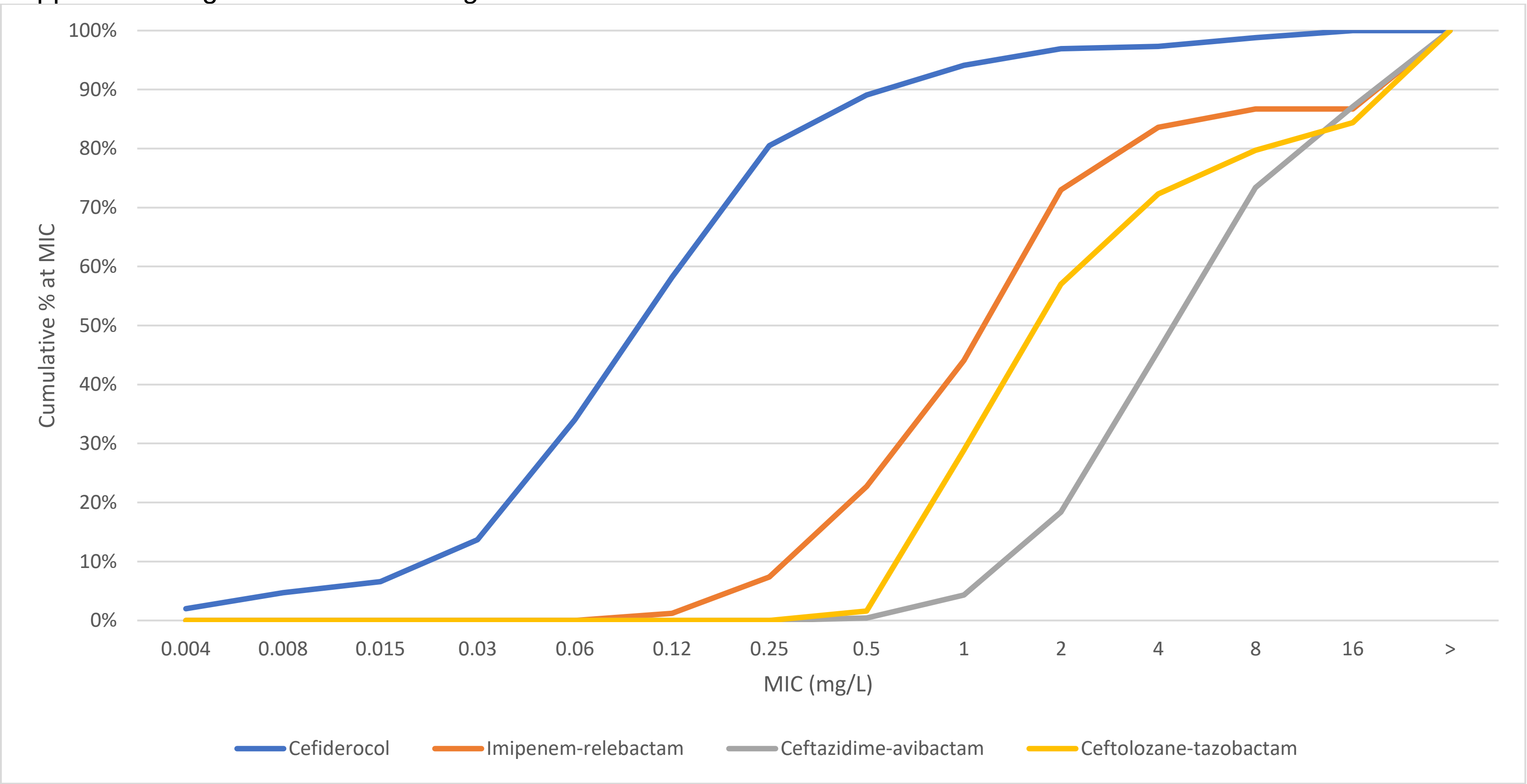

Supplemental figure 3. Meropenem-resistant *Acinetobacter* species

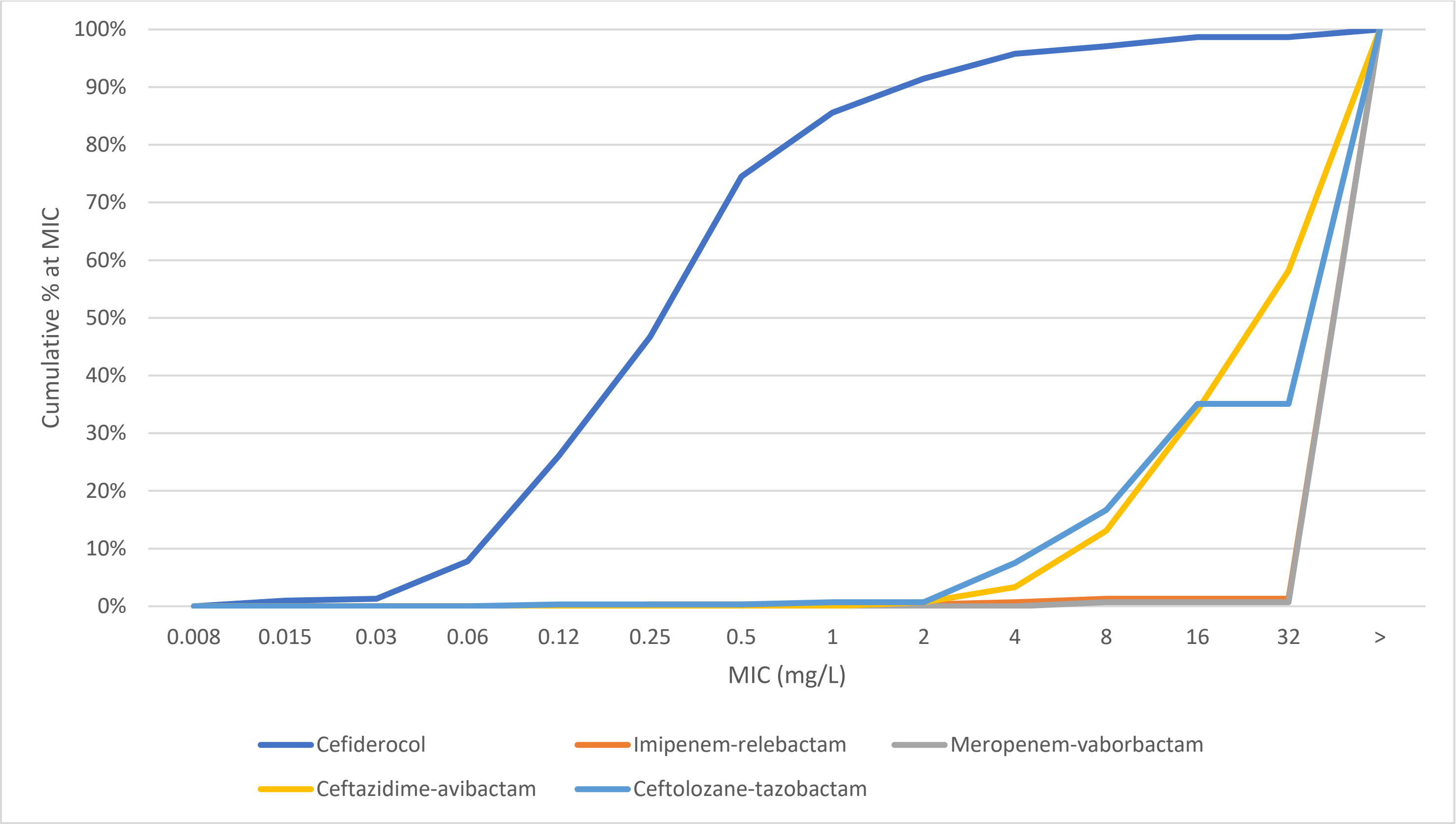

Supplement: SUPPLEMENTAL FILE 1 — Supplemental material. Download SPECTRUM02712-21_Supp_1_seq2.pdf, PDF file, 0.2 MB [file spectrum02712-21_supp_1_seq2.pdf]
